# Cool–Warm Temperature Stratification and Simulated Bird Digestion Optimize Removal of Dormancy in *Rosa rugosa* Seeds

**DOI:** 10.3389/fpls.2021.808206

**Published:** 2022-01-17

**Authors:** Peng Gao, Jie Dong, Sihan Wang, Wuhua Zhang, Tao Yang, Jinzhu Zhang, Daidi Che

**Affiliations:** ^1^College of Horticulture and Landscape Architecture, Northeast Agricultural University, Harbin, China; ^2^Key Laboratory of Cold Region Landscape Plants and Applications, Harbin, China; ^3^Aerospace Shenzhou Biotechnology Group Corporation Limited, Beijing, China

**Keywords:** Rosaceae, endangered species, seed dormancy, seed vitality, seed germination, temperature stratification, mutualism

## Abstract

*Rosa rugosa* Thunb. has been explored multi-function in medicinal, edible, cosmetic, ornamental and ecological etc. However, *R. rugosa* natural populations have recently declined substantially in China, besides of global climate change, this species also has the defect of limiting the reproduction of itself such as the hard-to-release seed dormancy. In this study, only 30% of *R. rugosa* seeds were viable, and the others were incompletely developed or diseased seeds. Without stratification, morphologically complete viable seeds imbibed water but those seeds could not germinate even after seed husk removal under suitable condition to exhibit a physiological dormancy. After cold (4°C) and warm (18 ± 2°C) stratification, macromolecular substances containing carbon or nitrogen accumulated, and respiration, antioxidant enzyme activity, and gibberellin (GA_3_) /abscisic acid (ABA) and auxin (IAA)/ABA ratios increased significantly in seeds. Water absorption also increased as endocarps softened. Thus, physiological dormancy of seed was broken. Although warm and cold stratification increased separation between endocarp and embryo, the endocarp binding force was removed insufficiently, because only 10.20% of seeds germinated. Therefore, stratified seeds were treated with simulated bird digestion. Then, folds and cracks in loosened endocarps increased permeability, and water absorption rate increased to 64.43% compare to 21.14% in cold and warm stratification treatment. With simulated digestion, 24.20% of radicles broke through the endocarp with plumules and cambiums to develop into seedlings. Thus, the seed dormancy type of *R. rugosa* is physiological as seeds imbibed water and possessed fully developed embryos with a low growth potential in combination with a mechanical constraint from the endocarp. Cold stratification helped remove physiological dormancy, and additional warm stratification accelerated the process. The optimal stratification treatment was 4°C for 45 days followed by 18 ± 2°C for 15 days. After warm and cold stratification, simulated bird digestion broke the mechanical constraint from the seed covering layers. Based on this research, production of *R. rugosa* seedlings can be greatly increased to help protect the species from further declines.

## Introduction

*Rosa rugosa* Thunb. from Rosaceae is a perennial ornamental shrub originally from east Asia (Yu et al., 1985). It produces beautiful flowers with a mild fragrance and colorful achenes. Unlike many other cultivated roses, *R. rugosa* is adapted to a wide range of habitat types and harsh environmental conditions, including salinity, alkalinity, shade, drought, high humidity, and frigid temperatures (Chen et al., 2021). *R. rugosa* is important in landscaping and because the fruit is rich in secondary metabolites and nutrients, as a raw material in food processing and medicine and cosmetics production (Chen et al., 2015). *R. rugosa* natural populations are generally distributed in dunes, where they have an essential role in maintaining coastal ecology (Qin et al., 1994). However, in recent decades, human activities have caused contrasting distributions of the species in China and some European countries. In northern Europe, *R. rugosa* is most likely one of the most frequent invasive plant species in natural and seminatural habitats (Jan et al., 2009). Growth and reproductive traits of populations of invasive *R. rugosa* from China are significantly better than those of native populations in coastal dunes of northwest Europe (Zhang et al., 2018). In particular, invasive *R. rugosa* have high and constant lateral clonal spread (Kollmann et al., 2009). By contrast, in China, natural *R. rugosa* populations have decreased sharply from year to year, and the species is now only distributed at the Tumen River in Jilin Province, on the southern coast of Liaoning Province, and on the eastern coast of Shandong Province (Li and Zhang, 2007). The natural habitat of *R. rugosa* has been greatly reduced in size and fragmented to such a degree that wild populations are in imminent danger of extinction (Yang et al., 2009). Therefore, the species is listed as an endangered plant in the China Plant Red Data Book (Fu, 1992). In nature reserves, growth of roses has improved, but how to expand and renew populations remains to be determined.

Although many clones can be produced through asexual reproduction in a short period, sexual reproduction and seed dispersal are the primary ways to maintain and regenerate plant populations (Myster, 2004; Johnson et al., 2020). In most woody plants in the Rosaceae, seed dormancy increases the time from fruit maturity to seed germination (Zhou and Bao, 2009). Because dormancy is a beneficial adaptation in intact natural systems that protects plants from germinating in non-growing seasons and harsh environments (Rubio de Casas et al., 2017), 50–90% of wild plant species worldwide produce seeds that are dormant at maturity. The five classes of dormancy are physiological, physical, combinational, morphological, and morphophysiological, and each requires specific cues to alleviate the dormancy and enable germination (Finch-Savage and Leubner-Metzger, 2006; Kildisheva et al., 2020). In Rosaceae, dormancy of apple (Daskalyuk, 2002) seeds is combinational (physical and physiological), while seeds of *Sorbus alnifolia* have a deep physiological dormancy (Tang et al., 2019), and germination increases with stratification or when growth regulators are used. With removal of physiological dormancy, seed respiration, cell differentiation and division, and concentration of gibberellin, which promotes germination, increase, whereas abscisic acid content, which inhibits germination, decreases (Finch-Savage and Leubner-Metzger, 2006). However, in *R. rugosa* seeds, dormancy type and how processes are affected when dormancy is removed are unclear. Mccully removed seeds from the feces of a dairy cow that was fed mature rose fruits and stratified them at 5°C for 2–18 months. Although rose pulp and endocarp softened, seed germination did not improve (Mccully, 1951). The results suggest that the release of rose seed dormancy may require a certain order and that removal of physiological dormancy before mechanical restriction would most benefit seed germination.

In China, the natural germination percentage of *R. rugosa* seeds is extremely low (Li and Zhang, 2007), and precise temperatures and times of stratification to promote rose seed germination are unknown. Thus, it is difficult to efficiently obtain sufficient numbers of rose seedlings. Plants have evolved delicious fruits to entice animals like birds into dispersing their seeds, and animals can help to establish and expand plant populations by digesting fruits, releasing seed dormancy, and spreading seeds over long distances after feeding (Gallagher, 2014). Dormancy and germinability of seeds are affected by which bird they pass through, with germinability of seeds generally increasing following gut passage in birds (Grime, 2001; Gallagher, 2014). Damage to seed coats in bird guts, particularly in their muscular gizzards, is often invoked to explain increased germination (Moore, 2001). Because of the bright color and high nutritional value, birds readily consume the fruits and seeds of *R. rugosa* (Møller et al., 2012; Martin-Albarracin et al., 2018). Therefore, the more underlying details can be critical for an in-depth understanding of how simulated birds feed and digestion operates within *R. rugosa* population to provide a comprehensive description of the molecular and physiological factors underlying dormancy among broad lineages of plants (Lennon et al., 2021).

In this study, *R. rugosa* seeds were collected from Hunchun, Jilin, China. To identify the type of seed dormancy, morphological and physiological changes were examined after stratification. Optimal stratification temperatures and times to release seed dormancy and germination of *R. rugosa* seeds after simulated bird digestion were determined. The focus of the research was to identify the type of *R. rugosa* seed dormancy, explore efficient methods to remove *R. rugosa* seed dormancy and promote as many healthy seeds as possible to germinate. By providing a theoretical basis for understanding *R. rugosa* seed dormancy and germination, the results can be used as reference to increase production of *R. rugosa* seedlings help protect the species from further declines in distribution and thus continue the development and utilization of this species by humans.

## Materials and Methods

### Plant Material

Fruits of *Rosa rugosa* Thunb. were harvested from a natural population in Hunchun City, Jilin Province, China (42°25′N, 130°03′E) in November of 2018 and 2019. Seeds were obtained by peeling off fruit flesh, naturally dried and stored in a refrigerator at 4°C.

### Stratification of *Rosa rugosa* Seeds

Fresh seeds packed in nylon mesh bags were covered with 5 cm of washed fine sand (40% water by volume) in 20-cm diameter × 10-cm deep pots. Pots were put into a refrigerator at 4°C (cold) or 18 ± 2°C (warm) in an artificial climate room (Shalimu et al., 2016). Figure 3A details stratification temperature and time treatments. Briefly, seeds were stratified under 4°C for 90 days in stratification treatment 1 (ST1) or under 4°C for 60 days and then were transformed into 18 ± 2°C for 20 days in ST2. In ST3, seeds were treated for 45 days at 4°C followed by 18 ± 2°C stratification for 45 days. In ST4, seeds were stratified under 18 ± 2°C for 60 days after 4°C cultivation for 30 days. In each treatment, seeds were stratified for 90 days and were collection at 0, 30, 60 or 90 days, respectively, for morphological observation and physiological index determination.

### Removing Endocarp Restraint on Embryos

After stratification (ST3), seeds were immersed in 98% H_2_SO_4_ for 5 min or gastric acid for 3 h. Seeds were pierced, scratched, or peeled in the groin by scalpel or dissecting needle to remove the mechanical bondage from endocarp (Supplementary Figure 1). Furthermore, seeds were soaked in ultrapure water and treated by an ultrasonic cleaner for 30 min (KQ5200DE, Shumei, Kunshan, China). To simulate bird consumption and digestion of seeds in glandular and muscular stomach (Zheng, 2012), stratified seeds (ST3) were soaked in an acidic solution (pH = 2.5) composed of gastric acid and HCl for 30 min. Then, seeds were placed in a shaking (60 turn/min) incubator at 42°C for 30 min. Gravel (1–2-mm diameter) were added to containers with seeds, followed by further shaking for 3 h.

All treated seeds were sown in absorbent cotton containing distilled water. Each treatment had 3 biological replicates, and in each replicate, germination of 60 seeds cultivated for 15 days was observed.

### Endocarp Characterization

A shore hardness (HSD) tester (LX-D, Syntek, Wuhan, China) was used to measure endocarp hardness; phloroglucinol staining was used to measure lignification of the endocarp; and a scanning electron microscope (TM3030, Hitachi, Tokyo, Japan) was used to observe seed surface and cross-sectional structure, according to the manufacturer’s instructions (Xiong et al., 2021). Endocarp permeability was indicated by the dyed area in endocarps immersed in methylene blue solution for 60 h. Dyed seeds were incised along the groin with a scalpel and observed under a stereomicroscope (SZX10, Olympus, Tokyo, Japan).

### Seed Vitality and Water Absorption

Seed embryos were split in half by scalpel along the axis and then incubated in 0.5% 2,3,5-triphenyltetrazolium chloride (TTC) solution (pH = 7.17) at 37°C for 1 h in the dark. Each treatment had three biological replicates with 60 seeds in each replicate. Seed viability was determined as follows:


S⁢e⁢e⁢d⁢v⁢i⁢a⁢b⁢i⁢l⁢i⁢t⁢y⁢p⁢e⁢r⁢c⁢e⁢n⁢t⁢a⁢g⁢e%=N⁢u⁢m⁢b⁢e⁢r⁢o⁢f⁢s⁢e⁢e⁢d⁢s⁢s⁢t⁢a⁢i⁢n⁢e⁢d⁢r⁢e⁢d/N⁢u⁢m⁢b⁢e⁢r⁢o⁢f⁢a⁢l⁢l⁢s⁢e⁢e⁢d⁢s× 100%

Seed water absorption was measured at 20–25°C. Initial weight (Wi) of 60 seeds was determined, and then, seeds were put into a 250-mL Erlenmeyer flask with 100 mL of distilled water. Seeds were removed at 0, 2, 4, 6, 8, 10, 12, 24, 36, 48,96, 192, 240 h, and final weight (Wf) was recorded. Each treatment had three biological replicates. Seed water absorption was calculated as follows:


Seedwaterabsorptionrate(%)=Wf-Wi/Wi×100%

### Physiological Indictors

Endogenous hormones gibberellin (GA_3_), abscisic acid (ABA) and auxin (IAA) were extracted and quantified according to the method of Cheng (Cheng et al., 2021). Starch granules were stained by iodine solution (1% KI and 0.5% I_2_)(Taiz and Zeiger, 2010). The anthrone colorimetric method was used to determine contents of soluble sugars and soluble starch, and Coomassie Brilliant Blue G-250 was used to determine soluble protein content (Taiz and Zeiger, 2010). Activities of peroxidase and superoxide dismutase enzymes and content of malondialdehyde were measured as described previously (Dong et al., 2021).

### Anatomical Observation of Seeds

To observe the germination process of *R. rugosa* seeds after removing the dormancy, seeds were put in a vial containing FAA fixative (70% alcohol:glacial acetic acid:formaldehyde = 90:5:5). A paraffin section preparation method was used to obtain sections (Wang et al., 2021), which were observed under an optical microscope (E200, Nikon, Tokyo, Japan). At each stage of development, more than 30 seeds were dissected.

### Statistical Analyses

Calculations were performed in Microsoft Excel 2016 (Redmond, WA, United States). All statistical analyses were performed using IBM SPSS v 22.0 software (Armonk, NY, United States), and the least significant difference (LSD) test in One-way ANOVA was used to test the difference among samples of each index under different treatments after the data were tested for normal distribution, equal variance and met the hypothesis requirements. Graphs and charts were prepared by using GraphPad Prism 8.0 (San Diego, CA, United States). Soluble sugars, starch, and protein contents were normalized as follows: *X*′ = *X*−*Xmin*/*Xmax*−*Xmin*, where *X* is the measured value, *Xmin* is the minimum value, and *Xmax* is the maximum value in the data set.

## Results and Analysis

### *Rosa rugosa* Seeds Enter Physiological Dormancy After Fruit Maturity

**FIGURE 1:**
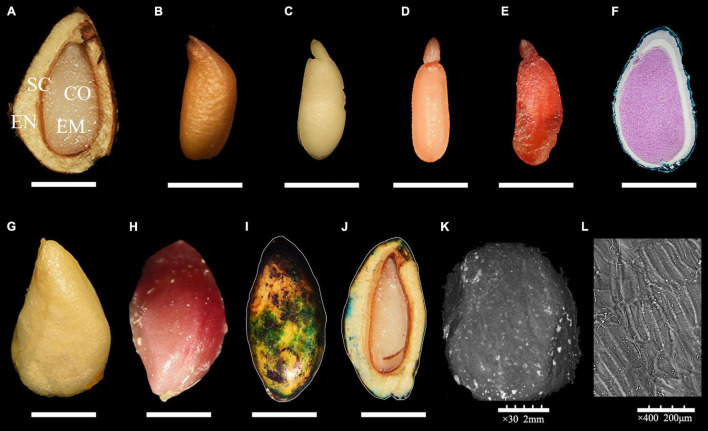
Morphology of *Rosa rugosa* seed. **(A)** Inside of seed in longitudinal section. Outer side of seed coat **(B)** and embryo in longitudinal section **(C)**. Bad **(D)** and healthy **(E)** embryos dyed with 0.5% 2,3,5-triphenyltetrazolium chloride (TTC) staining solution. **(F)** Rudimentary embryo. **(G)** Hard and thick endocarp **(G)** with lignin dyed red with phloroglucinol **(H)**. The outside **(I)** and inside **(J)** of the seeds that were penetrated by small volume of methylene blue solution. Scanning electron microscope observations of *R. rugosa* endocarp at 30 × **(K)** and 400 × **(L)**.

A complete *R. rugosa* seed was composed of lignified endocarp, seed coat, seed embryo and 2 cotyledons (Figure 1A). The endocarp was yellow and triangular in shape and had a tough and thick texture. The brown, membranous seed coat with longitudinal stripes (Figure 1B) was closely attached to the spoon-shaped seed embryo (Figure 1C). According to an earlier study, before fruit maturity, mature dicotyledon embryos are formed, and morphological development is complete (Wang et al., 2021). As fruit ripened, cell walls of the seed endocarp gradually thickened with increasing lignification to form a hard and smooth testa (Figures 1G,H) with a slow water absorption rate (Figures 1I,J and Supplementary Figure 2) and no obvious folds or cracks (Figures 1K,L). These changes can effectively protect an embryo from an unsuitable growth environment, but also limit the germination of seeds by reducing water absorption and gas exchange over a long period. In addition, among the randomly selected seeds, TCC dyed 30% seed embryos light red (Figure 1D) and therefore considered active (Figure 1E), the other seeds were too small, diseased to dark brown or the embryos are not fully developed (Figure 1F) even embryoless. However, these actively fresh embryos (without restraint from endocarp) failed to germinate even under suitable conditions. These results indicated that *R. rugosa* seeds entered a physiological dormancy with hard-to-break endocarp after fruit maturity.

### *Rosa rugosa* Seeds Reach Physiological Maturity After Cold Stratification

**FIGURE 2:**
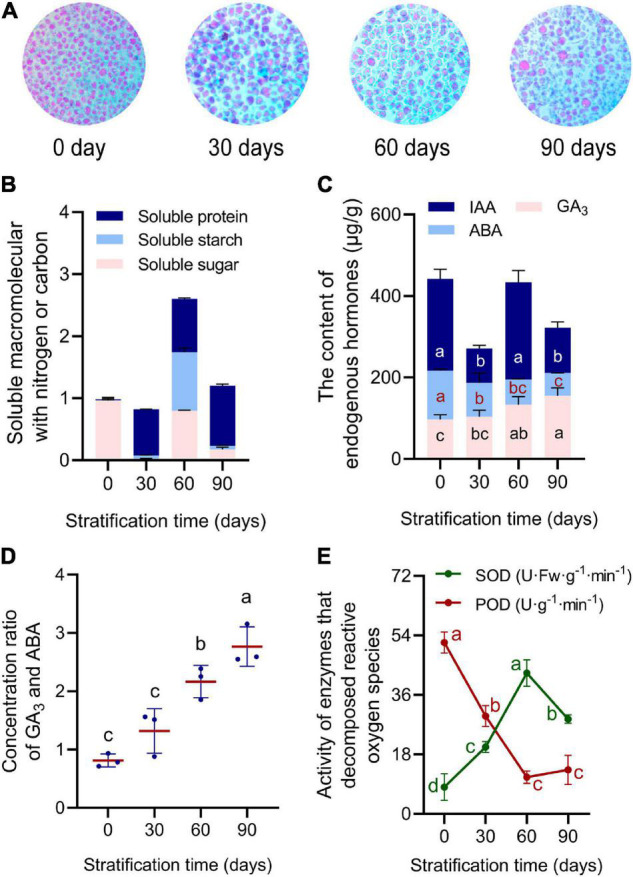
Physiological indicators in *Rosa rugosa* embryos were measured during stratification for 0, 30, 60, and 90 days at 4°C. **(A)** Size of starch granules increased and number decreased during stratification. **(B)** Soluble macromolecules (min–max normalized) with nitrogen or carbon peaked at 60 days of treatment. **(C)** Contents of Gibberellin (GA_3_) GA_3_ and Abscisic acid (ABA) changed linearly, whereas that of Auxin (IAA) changed in a waveform. **(D)** GA_3_/ABA ratio continued to increase. **(E)** Superoxide dismutase (SOD) and peroxidase (POD) activities showed opposite trends.

During 90 days of continuous cold stratification, physiological indicators in *R. rugosa* seeds changed significantly. Starch is an important energy source during seed dormancy and germination. In newly developed seeds, cotyledons were full of tightly packed similar-sized starch granules. As stratification time increased, numbers of starch granules decreased, and remaining granules had larger volumes and irregular shapes with obvious gaps among them (Figure 2A). Contents of soluble sugars and soluble starch in seeds changed in a zigzag-like pattern from 0 to 90 days of stratification, reaching peak values on day 60 of 68.19% and 1.14 mg/g, respectively (Figure 2B and Supplementary Figures 3A,B). Soluble protein content increased steadily, and reached the maximum value of 9.03 mg/g after 90 days of stratification (Figure 2B and Supplementary Figure 3C).

**FIGURE 3:**
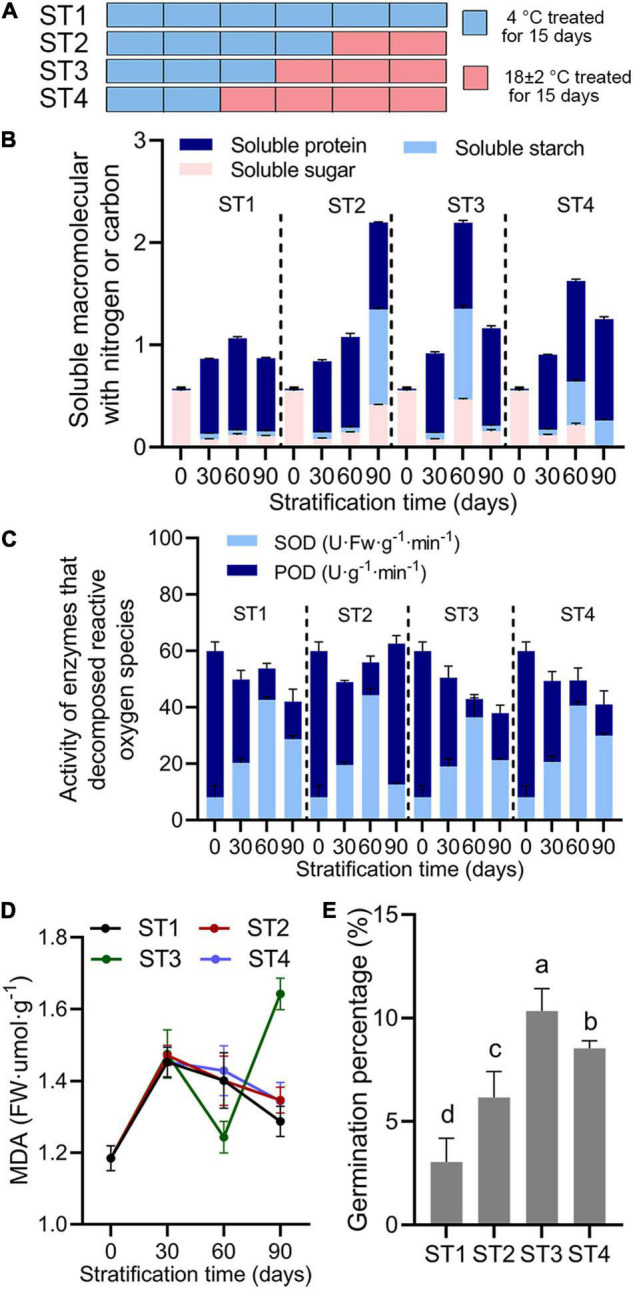
Physiological indices in *Rosa rugosa* seed embryos after alternate-temperature stratification. **(A)** Stratification treatments (ST) of seeds with different temperatures and times. Light blue bar indicates seeds were buried by sand at 4°C for 15 days, and peach bar represents treatment at 18 ± 2°C for 15 days. **(B)** Soluble protein, starch, and sugar contents, **(C)** peroxidase and superoxide dismutase activities, malondialdehyde content **(D)**, and seed germination percentage **(E)** among the four stratification treatments showed that embryo physiological activities were most active at 60 days in each stratification treatment. Seeds treated at 4°C for 45 days followed by 18 ± 2°C for 15 days (ST3) showed the best removal of physiological dormancy.

Content of gibberellin (GA_3_), which can promote seed germination, increased with stratification time, with the lowest content in untreated seeds (95.27 μg/g) and the highest content (155.10 μg/g) in seeds after 90 days of stratification (Figure 2C). By contrast, abscisic acid (ABA) content declined from 106.978 μg/g at day 0 to 56.929 μg/g after 90 days of stratification (Figure 2C). As a result, there was a gradual increase in the GA_3_/ABA ratio, which promoted removal of seed physiological dormancy (Figure 2D). Seed IAA levels fluctuated during stratification in an oblique “N”-like pattern, with the highest content of 204.22 μg/g at 60 days (Figure 2C). Superoxide dismutase (SOD) activity increased gradually and reached a maximum at 60 days of stratification (Figure 2E). Activity of peroxidase (POD) was highest from 0 to 30 days but then decreased to the lowest value at day 60 (Figure 2E). The changing physiological indicators demonstrated that seeds completed morphological development but did not reach physiological maturity when separated from the fruit. It could be inferred that the physiological dormancy of seeds was broken under cold stratification for 30 days. From day 30–60, soluble macromolecular substances containing carbon or nitrogen gradually transformed or decomposed to provide an energy source for the metabolic activities of seed germination. Furthermore, the GA_3_/ABA ratio increased gradually to release seed dormancy and promote seed germination, and embryos gradually matured from 60 to 90 days.

### Variable Temperature Stratification Increases Release of Physiological Dormancy in *Rosa rugosa* Seeds

Long-term stratification at 4°C gradually removed the physiological dormancy of embryos, and the addition of warm temperature stratification (Figure 3A) accelerated the process. Overall, soluble sugars and starch contents (Figure 3B and Supplementary Table 1), SOD activity (Figure 3C), and malondialdehyde content (Figure 3D) first increased and then decreased in stratification treatments (ST) 1–4. Soluble protein content continued to increase (Figure 3B and Supplementary Table 1) and peroxidase activity increased after a continuous decline in all treatments (Figure 3C). In each treatment, day 60 was the inflection point for each physiological index, which reached the peak or the maximum rate of change. Therefore, transformation to warm stratification after cold stratification benefited the accumulation of macromolecules with nitrogen or carbon in seed embryos and increased enzyme activity in order to reduce harmful substances in cells (Figures 3B–D). However, with continued stratification, aerobic respiration of embryos that had completed their physiological after-ripening was reduced, which was extremely unfavorable to seed development and germination (Figure 3E and Supplementary Table 1). These results suggested that 15–30 days of stratification at room temperature after 45–60 days of cold stratification released physiological dormancy of seeds to achieve higher germination percentage than other stratification treatment.

### Releasing Mechanical Constraints Is Necessary for Seed Germination

**FIGURE 4:**
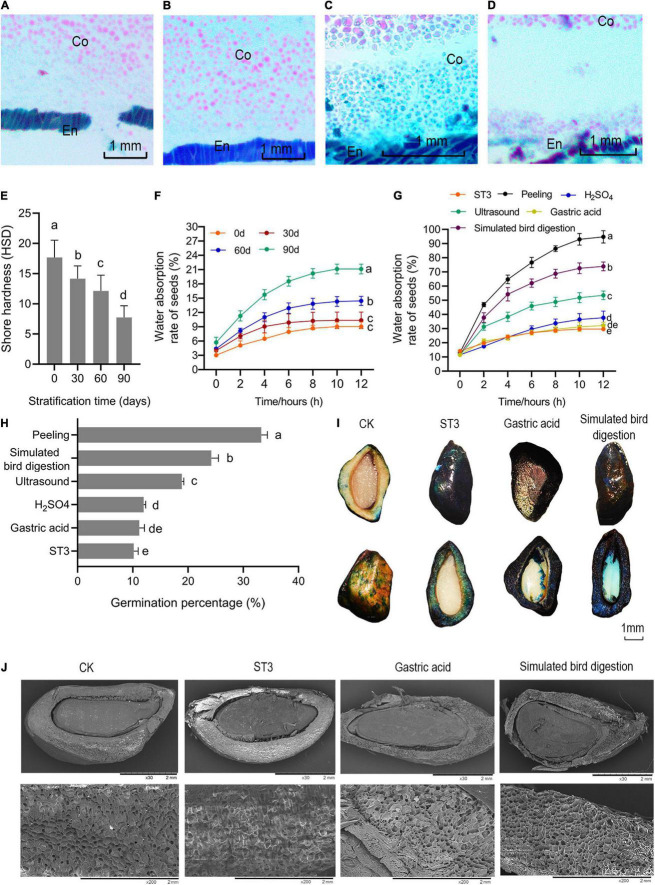
Releasing constraint by the endocarp on embryos promoted *Rosa rugosa* seed germination. Distance between endocarp and embryo increased gradually as stratification increased from 0 day **(A)** to 30 **(B)**, 60 **(C)**, and 90 days **(D)** in the ST3 treatment. **(E)** Seed hardness gradually decreased with the increase of stratification time. **(F)** Seed water absorption rate remained stable at 12 h in all treatments, and seeds with 90 days of stratification had the highest absorption. Seeds were treated with gastric acid, 98% H_2_SO_4_, simulated bird digestion, or peeling after ST3 treatment, and peeled seeds had the highest water absorption capacity **(G)** and germination percentage **(H)** followed by simulated bird digestion, which caused maximum penetration among chemical treatments with the largest area dyed blue **(I)** and maximum endocarp damage **(J)**. En, endocarp; Co, cotyledon.

The mechanical binding force of endocarp on seed embryos gradually weakened after stratification, but the change was not sufficient to allow most *R. rugosa* seed embryos to break through the endocarp. The endocarp tightly wrapped the seed embryo (Figure 4A) before ST3 treatment, and seed hardness was the highest at 17.67 HSD (Figure 4E). Subsequently, the long-term airtight and humid environment and friction of the sand softened and allowed infiltration of the endocarp. The binding force of endocarp to embryo apparently decreased as the separation between embryo and endocarp gradually increased, reaching the maximum separation on day 90 of stratification (Figures 4A–D). The softest endocarp occurred after 90 days (Figure 4E), and water absorption by seed embryos increased significantly (Figure 4F). However, the germination percentage of rose seeds remained low even after cold and warm stratification. When piercing, scratching, and peeling off the endocarp created a gradient of reduced binding force on embryos, seed water absorption and germination percentage increased and germination occurred earlier (Supplementary Figure 4). When the binding force was removed by peeling, water absorption by seed embryos suddenly increased, reaching 95% within 12 h. The germination percentage was as high as 33.33%, and speed of germination greatly improved with seeds germinating from the first day after planting. However, in addition to the inconvenience, manual husking of seeds greatly increases costs and can easily destroy the complete structure of seed embryos. Therefore, to identify a more efficient method, ultrasound, 98% H_2_SO_4_, gastric acid, and simulated bird digestion were used to release the restrict of endocarp for embryo that were stratified for 90 days (ST3). Acid etching did not increase seed germination, whereas ultrasound and simulated bird digestion significantly improved seed water absorption (Figure 4G) and germination percentage (Figure 4H). Ultrasound softened the endocarp and allowed penetration of cell membranes. When simulating the digestion process, the constant friction of pebbles on seeds increased water permeability of the endocarp (Figure 4I) and softened and thinned the seed husk, whose hard and dense surface loosened and became partly corroded with cracks (Figure 4J). As a result, seed germination percentage increased and was 2.37 and 7.96 times higher than that in ST3 and ST1, respectively. These results indicated that the hard and thick endocarp severely restricted water absorption by *R. rugosa* seeds even after 90 days of stratification, which limited germination of physiologically ripened seeds and restricted the extension of radicle and plumule. Simulated bird digestion efficiently and quickly thinned the testa without harming the activity of seed embryos and therefore was very effective at breaking the mechanical restriction of rose seeds covering.

### Germination Process of Rose Seeds After Release From Dormancy

**FIGURE 5:**
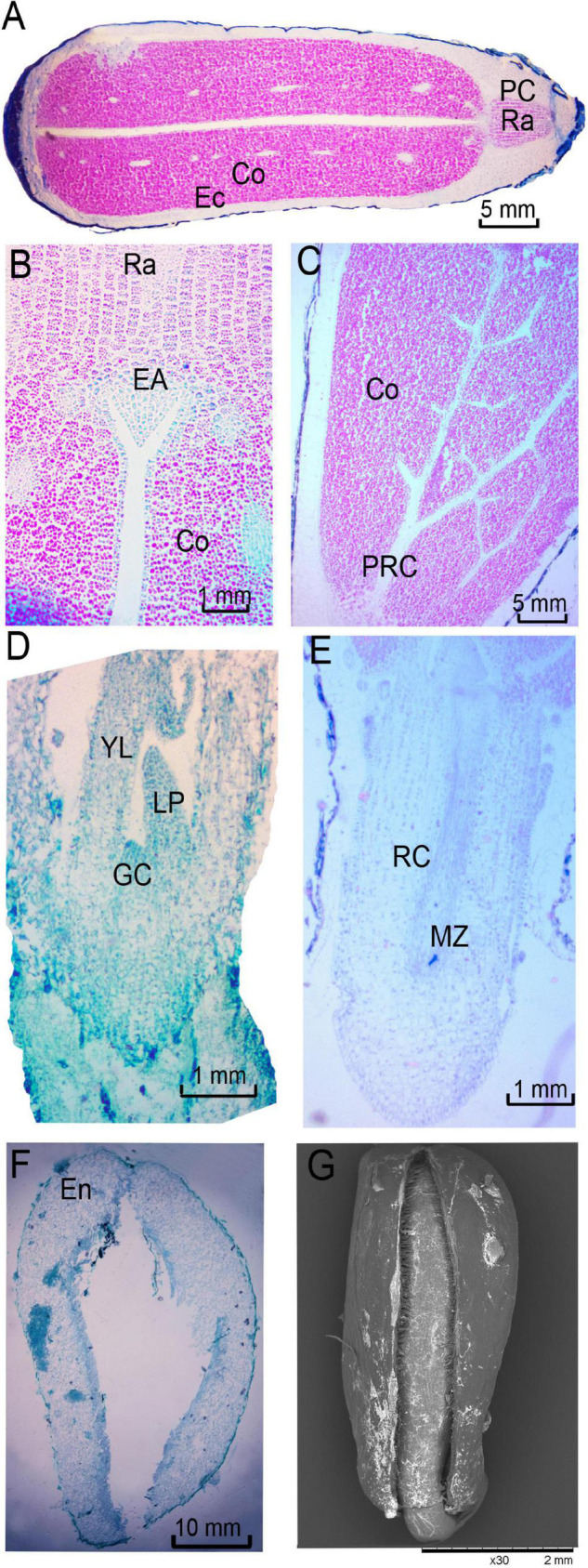
Anatomy of *Rosa rugosa* seeds after released from dormancy. **(A)** Exalbuminous seed with dicotyledon. **(B)** Triangular plumule primordium. **(C)** Protocambium. Developing plumule **(D)** and primary root **(E)**. **(F,G)** Endocarp after radicle breakthrough. EN, endocarp; CO, cotyledon; EC, ectocarp; RA, radicle; PC, parenchyma cells; EA, embryonic axis; GC, growth cone; PRC, protocambium; LP, leaf primordium; YL, young leaf; RC, root cap; MZ, meristem zone.

*Rosa rugosa* seeds treated with simulated bird digestion after stratification (ST3 treatment) began to germinate 6 days after sowing. As an exalbuminous seed (Figure 5A), *R. rugosa* embryos were composed of radicle, plumule, and cotyledons. There were two thickly fleshed cotyledons rich in starch grains, which provided the nutrients needed for later embryo growth (Figure 5A). The outermost parenchyma cells of cotyledons were tightly arranged with relatively small square shapes. Most inner parenchyma cells were tightly arranged in strips with relatively large round or polygonal shapes. After seeds were full of absorbed water, the meristematic cells at the radicle became larger with thicker cytoplasm as cells elongated and divided to form protrusions at the end of the radicle (Figures 5B,E). Ultimately, radicles broke through endocarps (Figures 5F,G). The plumule originated from the depression in the middle of the two cotyledons. It developed into a triangular-shaped convex embryo primordium (Figure 5B), followed by the development of growth cone, leaf primordium, and young leaves (Figure 5D). The protocambium in cotyledons differentiated into vascular tissue (Figure 5C), and there were 10–12 vascular bundles in the cross section that were distributed in the middle of cotyledons. Unfortunately, over 60% of *R. rugosa* seed embryos were incomplete or diseased

with low activity, and therefore, the probability was low that radicle and plumule could successfully break through even after manual intervention.

## Discussion

In nature, *R. rugosa* generally completes its growth cycle within 1 year, but the hard-to-break seed dormancy greatly limits this process (Righetti et al., 2015). A species invests in long-lived individuals (e.g., dormant seeds) to help populations persist through periods of unfavorable conditions (Lennon et al., 2021). Because most offspring develop close to their natal site, dormancy can alter classic metacommunity patterns of diversity in ways that depend on dispersal–dormancy covariation and spatiotemporal environmental variability (Wisnoski et al., 2019). In *Rosa* seeds, mechanical restriction is primarily caused by restriction by pericarp and testa, whereas physiological dormancy is due to inhibitory substances in pericarp, testa, and embryo (Zhou and Bao, 2009). Therefore, multiple methods are needed to break dormancy (Kildisheva et al., 2020). Gibberellin treatment does not break dormancy (Supplementary Figure 5), and several months of cold or warm stratification are required before derestriction and removal of inhibitory substances allow germination (Baskin and Baskin, 2004). Climate change may reduce the ability of this species to regenerate naturally and favor other species that require less chilling (Afroze and O’Reilly, 2013). Therefore, it is worth studying effect of the additional warm stratification and how long it is for plants that require long-term low-temperature stratification to break seed dormancy. For *R. rugosa*, the addition of room temperature stratification can promote the seeds to break dormancy and shorten the stratification time (Figure 3), but warm stratification time is not recommended to exceed 45 days, otherwise the seeds would germinate in the absence of light, low concentration oxygen, and malnutrition conditions, which backfire makes the seedlings unhealthy or even death.

Physiological dormancy is the most evolved and widely distributed dormancy type in seeds (Kildisheva et al., 2020), and it can be broken by regulating seed hormone levels and sensitivity, oxidative pathways, and metabolic activity. During seed maturation, ABA induces an adaptive trait called primary dormancy that prevents vivipary and after seed dispersal, delays and spreads germination over time (Sano and Marion-Poll, 2021). Endogenous ABA content of seeds reaches a peak during the green fruit period (Wang et al., 2021), causing physiological dormancy of seeds before they leave the fruit (Moore, 2001). As fruit matures, ABA content decreases, but seeds continue to have high sensitivity to ABA and maintain deep dormancy. After fruits leave the mother plant, seeds themselves continue to produce ABA during their development, increasing ABA content and imposing lasting dormancy (Kucera et al., 2005). Abscisic acid signaling is attenuated by ROS (reactive oxygen species) and NO (Nitric oxide) as seed coat integrity is lost, and seed oxygen content increases (Figure 2). With ABA degradation, dormancy is released by imbibition, which precedes activation of germination by GA (Finch-Savage and Leubner-Metzger, 2006). Maintaining dormancy also depends on a high ABA:GA ratio (Cadman et al., 2006). When ABA levels decrease, the root apical meristem produces GA, which stimulates water uptake through vacuolization that is combined with cell wall loosening of the hypocotyl and micropylar endosperm (Baskin and Baskin, 2004). Dormancy release involves a net shift to increased GA biosynthesis and ABA degradation that results in low ABA:GA ratios (Cadman et al., 2006). Ratios of GA/ABA, GA/IAA, and IAA/ABA and level of H_2_O_2_ also gradually increase in imbibed seeds with cold stratification, which in rice, may contribute to increased α-amylase activity that promotes dormancy release (Nagel et al., 2019). Furthermore, non-enzymatic reactions that remove germination inhibitors, reactive oxygen species and antioxidants (Bailly, 2004), membrane alterations (Hallett and Bewley, 2002), and degradation of specific proteins *via* proteasomes may also be important. In addition, compared with long-term cold stratification, 1 month of warm stratification plus cold stratification was superior in breaking the deep physiological dormancy of *Sorbus alnifolia* (Rosaceae) (Tang et al., 2019). *R. canina* seeds reach maximum percent germination under H_2_SO_4_ for 15 min followed by 4 weeks of warm stratification altered with 20 weeks of cold stratification (Iakovoglou and Radoglou, 2015). These results suggest that hormones may respond to environmental signals *via* changes in contents to transmit dormancy signals in processes of forming and removing seed dormancy. Seed respiration and antioxidant enzyme activities increased during release of dormancy, with new isoenzymes produced and consumption of stored macromolecular substances (Figures 2, 3). Then, new organs were produced as seeds germinated (Figure 5). In addition, thermal expansion and contraction of the endocarp caused by alternating cold and warm stratification accelerated cracking of the testa and increased embryo metabolism to release dormancy faster than that with only cold stratification (Shalimu et al., 2016).

Restraint by the endocarp or seed coat is the primary reason water absorption is inhibited in mature seeds, and simulated bird digestion effectively broke that cause of mechanical restriction. In seeds with coat dormancy, seed dormancy release and germination are determined by the balance of forces between the growth potential of the embryo and the constraint exerted by the covering layers (Kucera et al., 2005). Although ABA generally inhibits germination of seed embryos, it does not inhibit the rupture of testas (Finch-Savage and Leubner-Metzger, 2006). Seed transport in bird guts is a relatively effective mechanism to overcome the mobility problem faced by many plants. However, passing through an avian alimentary system can also affect overall viability of seeds as well as their dormancy characteristics and germination percentage (Moore, 2001). In this study, to simulate the digestion process of birds, the mechanical abrasion of the muscular stomach and digestion process of the glandular stomach were combined. The simulation improved seed germination percentage and delayed germination time compared with those under stratification treatment (ST3).

**FIGURE 6:**
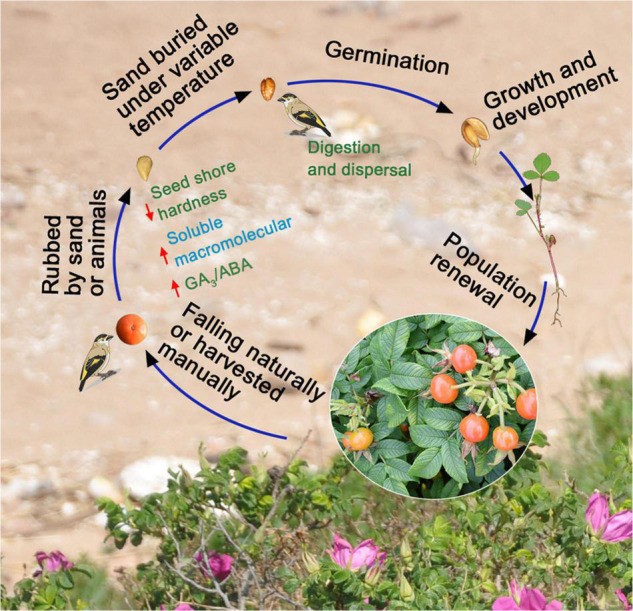
In nature, the possible process of seed harvest, stratification, germination, dispersal, and population update after the fruits of *Rosa rugosa* fall.

Plants and fruit-eating animals have established mutualistic relations to increase species survival and reproduction. Roses naturally spread over long distances, and seed germination is promoted *via* the feeding behavior of birds in different seasons (Figure 6). However, high-frequency human economic activities lead to sharp declines in numbers of wild animals and plants because of habitat fragmentation, which reduces communication between plants and animals and leads to decreases in biodiversity (Bruna et al., 2005). In Hunchun, populations of *R. rugosa* have gradually become patchy (Li and Zhang, 2007) and associated with the change in distribution, and greatly reduced numbers of birds (Yang et al., 2006) probably, which might restrict the dispersal and germination of *R. rugosa* seeds. Moreover, *R. rugosa* is completely gametophytic self-incompatible in China (Yu et al., 2009). This barrier to sexual reproduction reduces adaptability of the species and in combination with the hard-to-break seed dormancy and decrease in communication with animals, exacerbates endangerment of populations largely. Therefore, renewal of *R. rugosa* populations needs to be artificially regulated, in addition to establishment of the rose in nature reserves (Qu et al., 2011). To obtain high *R. rugosa* seed germination percentage, precise control of seed processing conditions and times with variable temperature stratification and simulated bird digestion are recommended. Whether mature fruits and seeds are harvested artificially or mechanically, after 45 days of cold stratification and 15 days of warm stratification to break physiological post-ripening dormancy, seeds and pebbles should be mixed to simulate the process of bird digestion. Last, seeds should be sown near *R. rugosa* populations to promote population growth and renewal (Figure 6). Next, whether there are differences in growth and ecological adaptability between artificially incubated seedlings and naturally germinated seedlings requires further observation and analysis.

## Data Availability Statement

The original contributions presented in the study are included in the article/Supplementary Material, further inquiries can be directed to the corresponding author/s.

## Author Contributions

DC and JZ conceived and designed the experiments. PG, JD, SW, WZ, and TY performed the experiments and interpreted the data. PG and JD analyzed the results and wrote the article. All authors read and approved the manuscript.

## Conflict of Interest

SW was employed by company Aerospace Shenzhou Biotechnology Group Corporation Limited. The remaining authors declare that the research was conducted in the absence of any commercial or financial relationships that could be construed as a potential conflict of interest.

## Publisher’s Note

All claims expressed in this article are solely those of the authors and do not necessarily represent those of their affiliated organizations, or those of the publisher, the editors and the reviewers. Any product that may be evaluated in this article, or claim that may be made by its manufacturer, is not guaranteed or endorsed by the publisher.

## Supplementary Material

Supplementary Figure 1 Methods of mechanical scarification. Front view **(A)** and side view **(B)** of seed with full endocarp, scratching **(C)**, piercing **(D)**, or **(E)** peeling of seed endocarps.

Supplementary Figure 2 After natural drying, the water absorption rate of intact seeds or seeds with endocarp removed was investigated.

Supplementary Figure 3 Soluble sugar **(A)**, starch **(B)**, and protein contents **(C)** of embryos during stratification for 0, 30, 60, and 90 days at 4°C.

Supplementary Figure 4 Seed germination percentage were calculated without damage or with scratching, piercing, or peeling of seed endocarps.

Supplementary Figure 5 Seed germination percentages were calculated after exogenous GA_3_ treatment.

Supplementary Table 1 Physiological indices of *Rosa rugosa* seeds under alternate-temperature stratification.
