# Supplementary figures and images for: Cool–Warm Temperature Stratification and Simulated Bird Digestion Optimize Removal of Dormancy in Rosa rugosa Seeds
Source: Front Plant Sci. 2022 Jan 17;12:808206. doi: 10.3389/fpls.2021.808206 (PMC8801612; doi:10.3389/fpls.2021.808206)

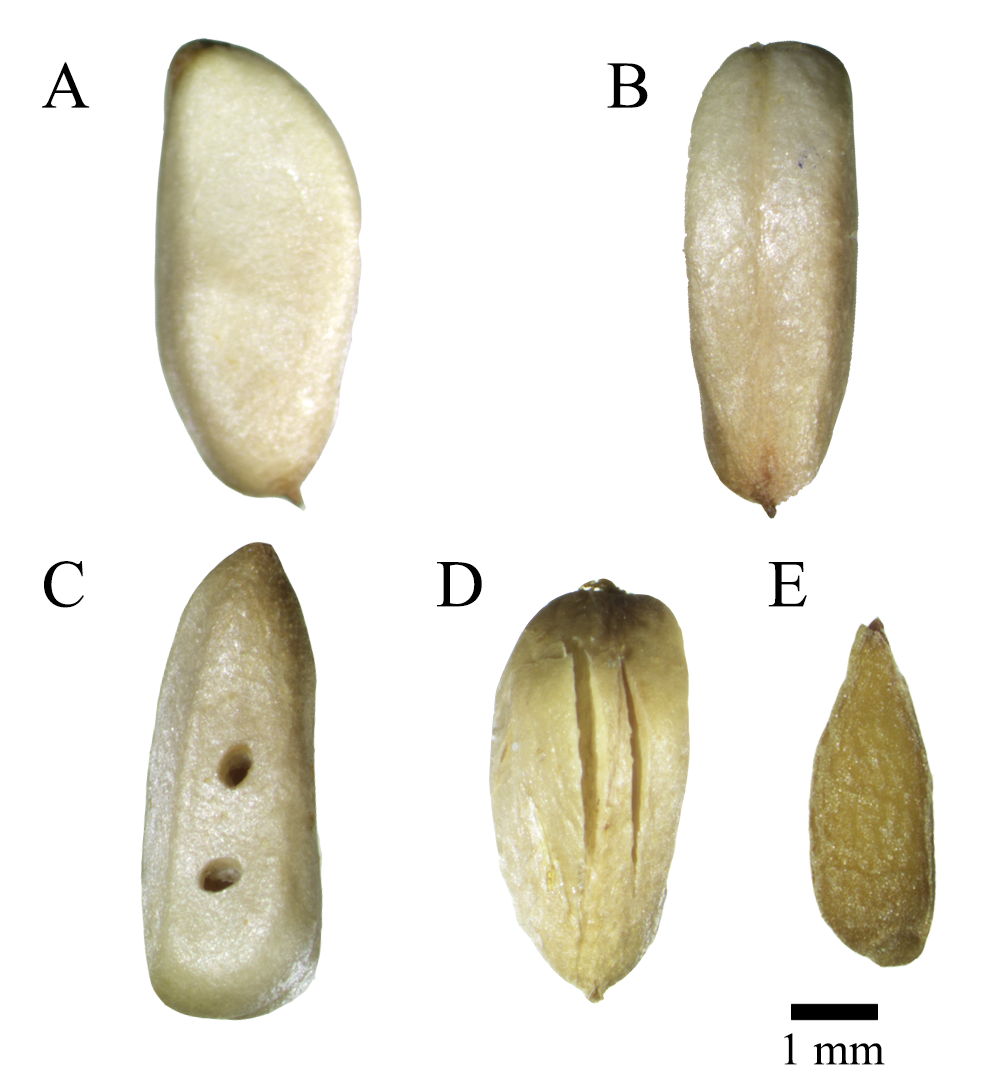

Supplement: Supplementary Figure 1 — Methods of mechanical scarification. Front view (A) and side view (B) of seed with full endocarp, scratching (C), piercing (D), or (E) peeling of seed endocarps. [file Image_1.TIF]

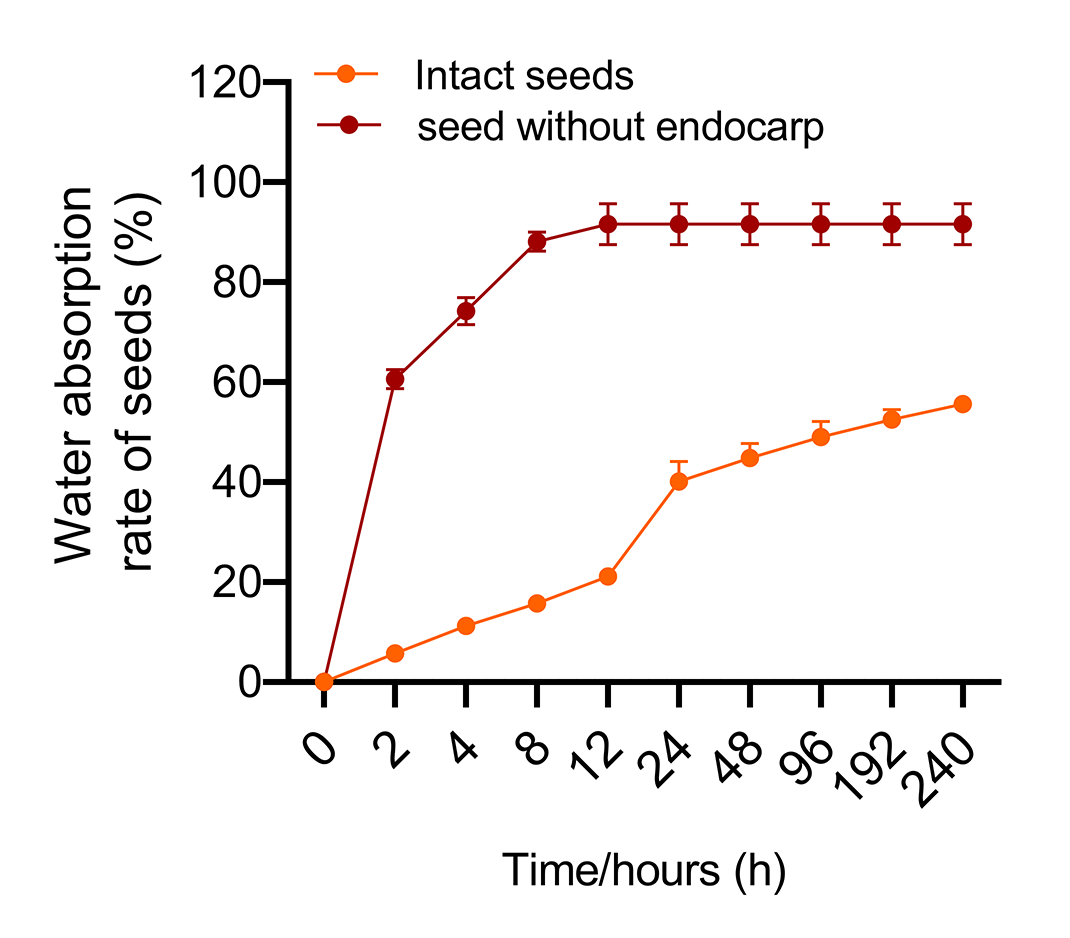

Supplement: Supplementary Figure 2 — After natural drying, the water absorption rate of intact seeds or seeds with endocarp removed was investigated. [file Image_2.TIF]

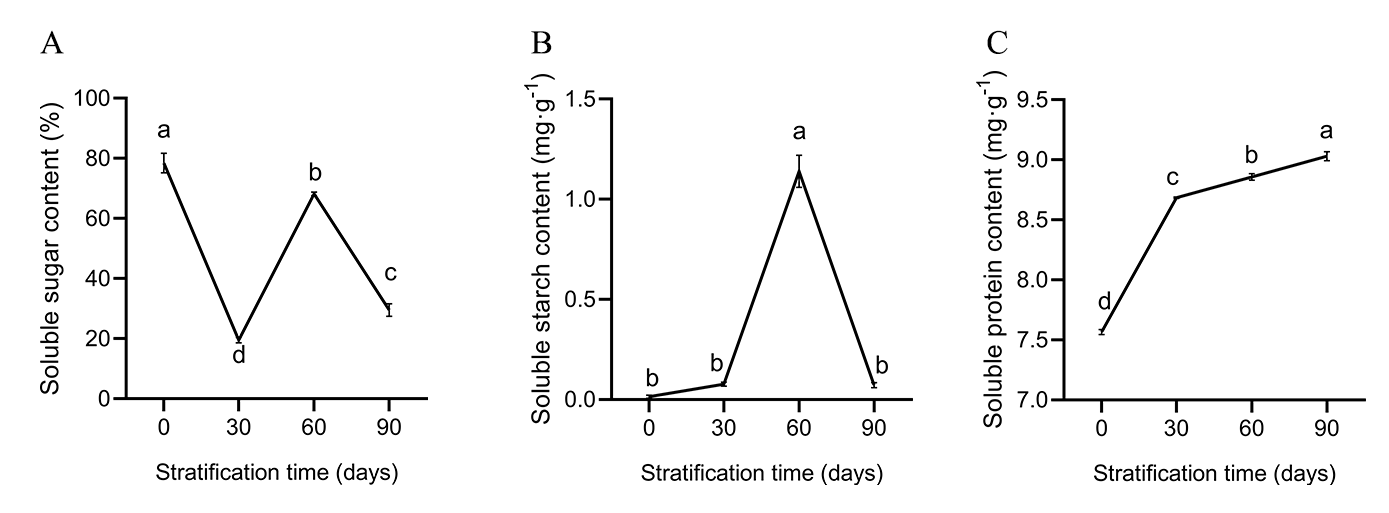

Supplement: Supplementary Figure 3 — Soluble sugar (A), starch (B), and protein contents (C) of embryos during stratification for 0, 30, 60, and 90 days at 4°C. [file Image_3.TIF]

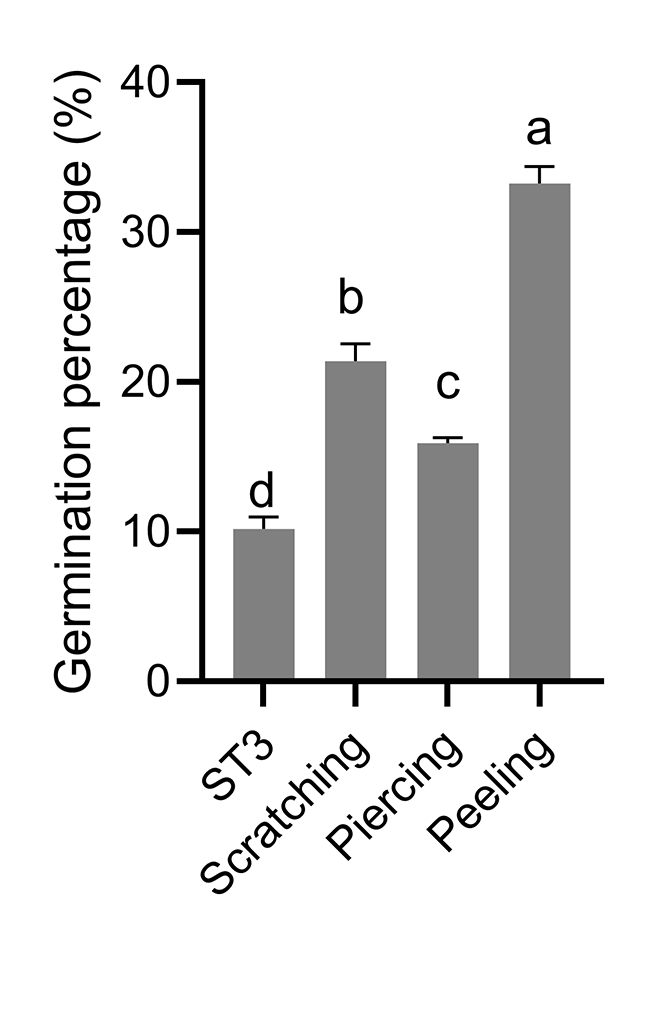

Supplement: Supplementary Figure 4 — Seed germination percentage were calculated without damage or with scratching, piercing, or peeling of seed endocarps. [file Image_4.TIF]

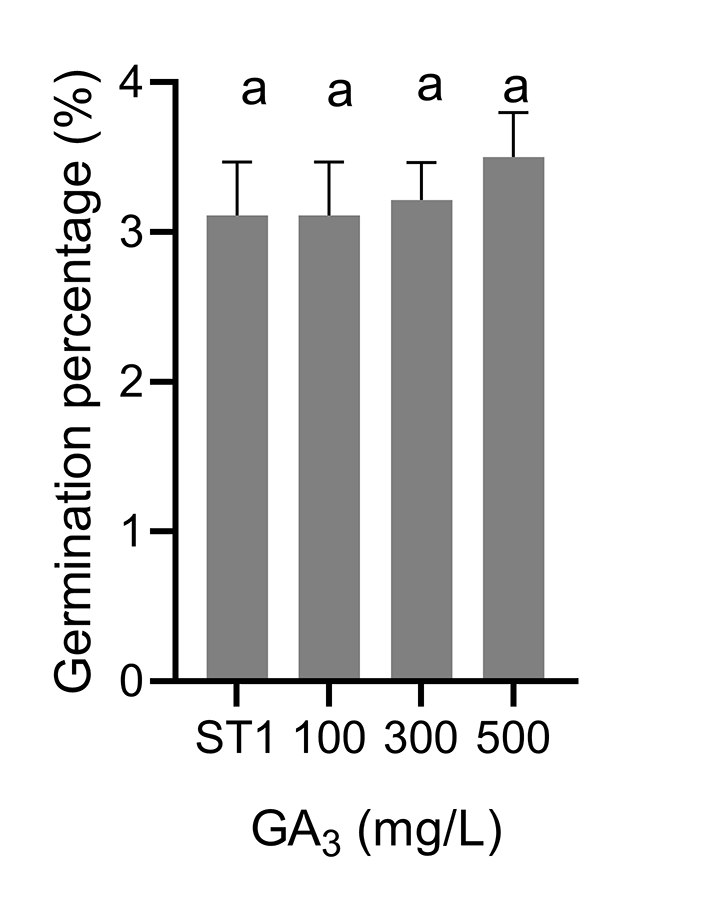

Supplement: Supplementary Figure 5 — Seed germination percentages were calculated after exogenous GA3 treatment. [file Image_5.TIF]
